# Scientific Items

**Published:** 1883-10-20

**Authors:** 


### SCIENTIFIC ITEMS.

**Limits of Germination.**—Pasteur, in his investigations regarding fermenting germs and bacteria, demonstrated that a temperature of 212° Fahr. was sufficient to destroy the power of germination in these minute forms of animal and vegetable life; but with the higher orders of plant and animal life, a much lower heat than 212° will destroy all vitality. According to Goppert. 30° F. is the lowest temperature at which most seeds would germinate.

The following table by Sach shows the lowest, the highest and most favorable temperature for germination of wheat, corn and barley:

| | Lowest Temp. | Highest Temp. | Temperature of Rapid Germination. |
|---|---|---|---|
| Wheat | 41° F. | 104° F. | 84° F. |
| Corn | 41° | 104° | 84° |
| Barley | 48° | 115° | 93° |

Experiments by the writer to verify the germinating point for wheat, revealed the fact that the temperature given is too low; as, in one instance, wheat that had been heated to 130° F. germinated. The grains were sprouted on cotton in the open air, and not in the laboratory by artificial heat; and the July sun and different wheat may explain this difference in results.

The time required for germination varies—with wheat, rye, corn and oats three to six days being sufficient.—*Mechanical News.*

**First Idea of the Telephone.**—The following lines, brought to the attention of M. de Parville, by Prof. Egger, are extracted from the book "Incredulite et Mescreance du Sortilege," by P. de l'Ancre, published at Paris, in 1662. De la Divination, 5 teme traite:

"It is reported that a German communicated to King Henry the Great an astonishing secret, through which men far apart might understand each other by means of the magnet. He first rubbed together (frotta) two magnetic needles, and then attached them separately to two clocks, about the dials of which were written the twenty-four letters of the alphabet. When one needle was moved to a letter of the alphabet the other, no matter how far away, moved to the same letter. The king perceiving how dangerous the secret might become in transmitting information to besieged cities, forbade its publication."—*Med. and Surg. Rep.*

**Shortness of Time in Dreams.**—One of the most remarkable phenomena connected with dreams is the shortness of time needed for their consummation. Lord Brougham says, that, in dictating, a man may frequently fall asleep after uttering a few words, and be awakened by the amanuensis repeating the last word to show he has written the whole. But, though five or six seconds only have elapsed between the delivery of the sentence and its transfer to paper, the sleeper may have passed through a dream extending through half a life-time. Lord Holland and Mr.

Babbage both confirm this theory. The one was listening to a friend reading aloud, and slept from the beginning of one sentence to the latter part of the sentence immediately succeeding; yet, during this time he had a dream, the particulars of which would have taken more than a quarter of an hour to write. Mr. Babbage dreamed a succession of events, and woke in time to hear the conculding words of a friend's answer to a question he had just put to him. One man was liable to a feeling of suffocation, accompanied by a dream of a skeleton grasping his throat, whenever he slept in a lying posture, and had an attendant to wake him the moment he sank down. But, though awakened the moment he began to sink, that time sufficed for a long struggle with the skeleton. Another man dreamed that he crossed the Atlantic, spent a fortnight in America, and fell overboard when embarking to return; yet his sleep had not lasted more than ten minutes.—*Pop. Sci. News.*

**Blood Poison.**—Wm. C. Conant in a paper read May 10th, 1883, before the Polytechnic Association of the American Institute, says:

"The most wonderful achievement of recent investigation reveals a philosophy of both bane and antidote that astonishes us with its simplicity as much as with its efficiency. At the moment when humanity stands aghast at the announcement that germs are not destroyed by disinfectants, comes the counter discovery that they are rendered harmless by oxygen. It seems that it makes no difference, really, of what sort or from what source are the bacteria that we take into the blood. The only material difference to us depends on the sort of atmosphere in which their hourly generations are bred. For example, the bacteria developed in confined air, from a simple infusion of hay, are found by experiment to be as capable of generating that most terrible of blood-poisoners, the malignant pustule, as are the bacteria taken from the pustule itself.

"On the other hand, the bacteria from the malignant pustule itself, after propagating for a few hours in pure and free air, become a perfectly harmless race, and are actually injected into the blood with impunity. The explanation of the strange discovery is this —note its extreme simplicity—bacteria bred in copious oxygen perish for want of it as soon as they enter the blood-vessels; whereas those inured to an unventilated atmosphere for a few generations, which means only a few hours, are prepared to thrive and propagate infinitely within our veins; and that is the whole mystery of blood-poisoning and zymotic diseases."

**According** to Electricite, spiders, which are very numerous in Japan, spin their webs during the night, between the telegraph wires and their supports. As the dews are very abundant, the webs become conductors of electricity and give rise to great disturbance in the transmission of messages.—*Ex.*

